# Phenotypic Buffering in a Monogenean: Canalization and Developmental Stability in Shape and Size of the Haptoral Anchors of *Ligophorus cephali* (Monogenea: Dactylogyridae)

**DOI:** 10.1371/journal.pone.0142365

**Published:** 2015-11-06

**Authors:** Cristina Llopis-Belenguer, Juan Antonio Balbuena, Iván Galván-Femenía, Abril Rodríguez-González

**Affiliations:** 1 Marine Zoology Unit, Cavanilles Institute of Biodiversity and Evolutionary Biology, University of Valencia, Valencia, Spain; 2 Department of Computer Science, Applied Mathematics and Statistics, Universitat de Girona, Girona, Spain; Fred Hutchinson Cancer Research Center, UNITED STATES

## Abstract

Phenotypic variation results from the balance between sources of variation and counteracting regulatory mechanisms. Canalization and developmental stability are two such mechanisms, acting at two different levels of regulation. The issue of whether or not they act concurrently as a common developmental buffering capacity has been subject to debate. We used geometric morphometrics to quantify the mechanisms that guarantee phenotypic constancy in the haptoral anchors of *Ligophorus cephali*. Canalization and developmental stability were appraised by estimating inter- and intra-individual variation, respectively, in size and shape of dorsal and ventral anchors. The latter variation was estimated as fluctuating asymmetry (FA) between anchor pairs. The general-buffering-capacity hypothesis was tested by two different methods based on correlations and Principal Components Analyses of the different components of size and shape variation. Evidence for FA in the dorsal and ventral anchors in both shape and size was found. Our analyses supported the hypothesis of a general developmental buffering capacity. The evidence was more compelling for shape than for size and, particularly, for the ventral anchors than for the dorsal ones. These results are in line with previous studies of dactylogyrids suggesting that ventral anchors secure a firmer, more permanent attachment, whereas dorsal anchors are more mobile. Because fixation to the host is crucial for survival in ectoparasites, we suggest that homeostatic development of the ventral anchors has been promoted to ensure the morphological constancy required for efficient attachment. Geometric morphometrics can be readily applied to other host-monogenean models, affording not only to disentangle the effects of canalization and developmental stability, as shown herein, but to further partition the environmental and genetic components of the former.

## Introduction

The phenotypic variation found in organisms is only a subset of all possible phenotypes because the developmental process limits the range of phenotypic variability. This subset is what is available to natural selection and biases the developmental process allowing evolution in future generations [1–3]. It is widely accepted that phenotypic variation results from the balance between sources of variation, such as genetic mutations, environmental effects and developmental errors; and counteracting regulatory processes buffering against this variation [4]. Although the distinction between the different regulatory mechanisms is somewhat blurred and is still subject to debate [4, 5], canalization and developmental stability are often considered as evolutionary processes acting at two different levels of regulation [4, 6, 7]. The former is viewed as a process that buffers against genetic and environmental perturbations avoiding the production of unexpected phenotypes. Developmental stability, in turn, would buffer random developmental errors, intrinsic to the cellular processes responsible for development of morphological structures [4, 6–8]. The question whether canalization and developmental stability represent two distinct processes can be tackled by comparing inter-individual variation within a population with intra-individual variation [6–8].

Because buffering mechanisms control the expression of phenotypic variation, understanding their functioning is a fundamental challenge in evo-devo [9]. It has been hypothesized that mechanisms that maintain the phenotypic stability of a trait against one type of genetic or environmental perturbation will protect the trait against all other types of perturbation [10, 11]. Thus, a number of studies have endeavoured to establish whether there is such as general developmental buffering capacity, including canalization and developmental stability, or, alternatively, whether individual variation is buffered independently by each of these regulatory mechanisms [12]. Hsp90 protein has been recognised as a common determinant of different pathways buffering both genetic and environmental perturbations [13]. In this context, geometric morphometrics (GM) has proven to provide a reliable framework to study the relationship and disentangle de effects of canalization and developmental stability [6, 7, 11, 14]. A decisive advantage of this approach is that it facilitates separating inter-individual from intra-individual variation. The latter can be measured as the fluctuating asymmetry (FA) of bilaterally symmetric traits [6]. Under this approach, the key assumption is that each side of the organism or structure under study represents a replicate expression of a single genotype in a single environment. Thus, right-left (R-L) differences should reflect the intrinsic variance of the developmental process (intra-individual variation) and thus FA estimates are expected to be inversely proportional to developmental stability [6, 15–17].

GM approaches have been proven to be fruitful in a wide range of model organisms and characters [16, 18, 19], but it has seldom, if ever, been applied to helminths and other parasitic organisms [20, 21]. Monogenea represent a class of Platyhelminthes. They are mostly external parasites on the skin and gills of fish and have direct life cycles [22]. GM studies can be particularly valuable in monogeneans with bilaterally symmetrical anchors. Given that anchors are essential for host exploitation in monogeneans [23, 24], it can be hypothesised that mechanisms buffering against morphological variation in anchors leading to suboptimal exploitation exist. Consequently, determining how genetic, environmental and developmental perturbations are buffered can make fundamental contributions to the understudied area of evolutionary developmental biology of helminths [21].

**Fig 1:**
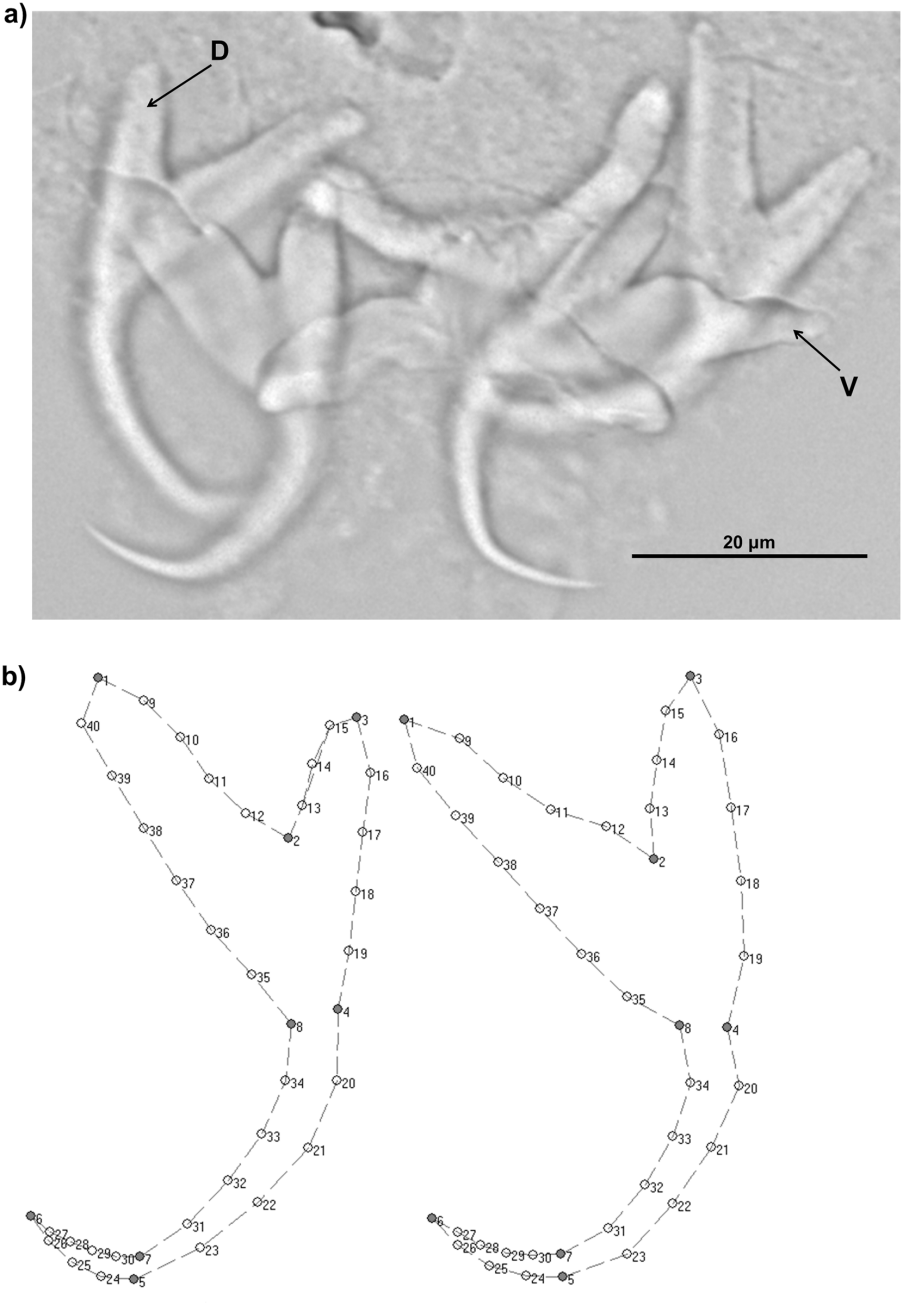
Haptoral elements of *Ligophorus cephali*. (A) Microscopic photography of dorsal and ventral anchors with their respective bars. (B) Distribution of landmarks (1–8, filled points) and semilandmarks (9–40, open points) considered in the present study in dorsal and ventral anchors. Landmarks were defined as follows: 1, top of inner root; 2, inflexion between outer root and inner root; 3, top of outer root; 4, outer shaft base; 5, outer point base; 6, tip of point; 7, inner point base; 8, inner shaft base. Groups of three to six semilandmarks were placed equidistantly between landmark pairs as shown.

The present study focuses on the analysis of inter- and intra-individual (FA) variation in the shape and size of the sclerotized haptoral anchors of *Ligophorus cephali* Rubtsova, Balbuena, Sarabeev, Blasco-Costa et Euzet, 2006. The haptor in *Ligophorus* spp. consists of seven pairs of marginal hooks and two pairs of anchors (dorsal and ventral), which are connected by respective transversal dorsal and ventral bars (Fig 1A) [25]. *Ligophorus cephali* is a good model for inter- and intra- individual variation studies because thanks to the symmetry of their hard anchors intra- and inter- individual variation can be easily measurable. In contrast, this type of studies would be more difficult to perform with other parasite groups that lack sclerotized structures.

Our general objective was to evaluate the mechanisms buffering canalization and developmental stability of the haptoral anchors of *L*. *cephali*. Given that in *Ligophorus*, as in other Dactylogyridae, the haptor exhibits two pairs of anchors (ventral and dorsal), comparison of variation patterns between them affords the formulation of more elaborate hypotheses than in most previous studies where only one set of structures (matching symmetry) or a single bilaterally symmetric structure (object symmetry) is the subject under investigation [12]. Our specific goals were (1) to test the hypothesis that there is a common mechanism buffering phenotypes against development instability and environmental perturbations; (2) establish whether morphological variation in anchor shape and size is buffered separately; and (3) evaluate potential differences in buffering mechanisms between dorsal and ventral anchors. The evidence presented herein points to differences in buffering processes between dorsal and ventral anchors and a general mechanism buffering against phenotypic variation in shape of anchors. These patterns are justified in terms of putative functional differences in attachment between dorsal and ventral anchors.

## Materials and Methods

### Ethic statement

The fish was obtained within day-to-day fishery operations and purchased dead from licensed commercial fishermen. The number of specimens used in the study (31) was kept to a reasonable minimum to guarantee the success of the research. Flathead grey mullets (*Mugil cephalus*) are both locally and globally abundant and are not subjected to special conservation regulations in Spain. The species is listed by the UICN as “Least Concern”.

### Study

Fish required for this study were obtained within normal fishery operations. Flathead grey mullets (*Mugil cephalus* L.) were purchased from a local fishing community licensed by the Department of Agriculture, Fishing, Food and Water of the Generalitat Valenciana. The fish was captured in L’Albufera (39° 20’ N—0° 21’ W) (Spain), a shallow and highly eutrophicated, 23.2 km^2^ Mediterranean lagoon [26, 27]. Thirty-one specimens were collected in a single locality (El Palmar, Valencia) within one day. Examination of the gills revealed the presence of 325 monogean specimens that were identified as *L*. *cephali* following Rubtsova et al. [28]. Among them, 35 individuals from seven hosts were selected for the present study because at least a pair of anchors of the haptor was present, visible in all planes and without apparent deformation for morphometric analyses (Fig 1A). The transversal bars, dorsal and ventral, were not considered for study because they support the compression effort and they are more susceptible to deformation than the anchors [29].

In order to facilitate the observation of the anchors soft tissues were degraded by means of an enzymatic digestion technique as described in Rodríguez-González et al. [21]. The anchors were then drawn using a drawing tube at 100× (under oil immersion) in the Nikon Optiphot-2 microscope fitted with interference contrast. The drawings were digitized to create independent images for each anchor.

We used GM to evaluate the symmetry patterns of the anchors. This technique is based on spatial coordinates of landmarks that are placed over an image of the organism or structure under study [30]. Dorsal and ventral pairs of anchors were processed independently. In each anchor eight landmarks, whose homology was easily recognizable, were placed [21] (Fig 1B). In addition, in order to get a more accurate description of the anchor morphology, we employed semilandmarks [31]. This is appropriate in our case because curves of roots, blade and point lack of easily detectable homologous points but differences in the curvature between sides captured by semilandmarks can be biologically relevant [20, 32], particularly when evaluating intra-individual differences, which a priori are expected to be subtle. Eight groups of three to six semilandmarks were placed equidistantly between the landmark pairs as shown in Fig 1B. Hence, the morphology of each anchor was defined by the Cartesian coordinates (*x*, *y*) of the 40 anatomical points (i.e. landmarks and semilandmarks). These geometric coordinates were processed with the TPS series [33]. Furthermore, we used this package to estimate anchor size as centroid size (square root of the sum of squared distances of a set of landmarks from their barycentre) [34].

In order to account for the error incurred in drawing, image acquisition and placing of the landmarks [15, 16, 19], all anchors were processed twice by a different researcher, rendering two replicates of the dataset for the subsequent statistical analysis [16, 19, 34].

We performed a Procrustes analysis with MorphoJ 1.06e [35] to separate shape from general information about size and other parameters (such as position and orientation) of each anchor [31]. This method consists of superimposing two or more configurations of anatomical points in order to obtain their consensus shape [16, 34]. Procrustes ANOVA was used to evaluate differences in shape between the left and right anchors with the Procrustes coordinates of landmarks and semilandmarks [32, 34]. Likewise, two-way ANOVA was employed to test the corresponding differences in size. These analyses were performed independently for dorsal (*N* = 25) and ventral (*N* = 30) anchors. In both statistical settings, the factors considered were individual (specimen of *L*. *cephali* as a random factor), side (left and right as fixed effects), the interaction between individual and side, and replicate (to take experimental error into account). Both the Procrustes and two-way ANOVAs were carried out with MorphoJ 1.06e [35].

In order to give a proper interpretation of the interaction term, in both Procrustes and two-way ANOVA, four additional statistical submodels were performed without the interaction term. These models were tested by using the *adonis*() function in the R package **vegan** [36] for the Procrustes ANOVA and the *aov*() function from the R package **stats** [37] for the two-way ANOVA.

Concerning the assumptions of the Procrustes ANOVA and two-way ANOVA, individuals are independent observations because they came from a random sample of fish in a given locality in a given time. Normality was not tested for the Procrustes ANOVA due to the fact that it is a multivariate non-parametric test [38, 39]; whereas, normality was tested for the replicate in the two-way ANOVA using the *shapiro*.*test*() function from the R package **stats** [37]. Finally, homoscedasticity was tested for the factor side using the function *betadisper*() in the R package **vegan** [36] in the Procrustes ANOVA; and using the function *leveneTest*() from the R package **car** [37] for the two-way ANOVA.

Given that both analyses produce the same statistical output for FA and antisymmetry, we inspected the Principal Component Analyses (PCA) plots of the Cartesian coordinates of landmarks (shape) [16, 34] and the frequency distribution of the signed R-L differences of anchor size [15] to eventually discriminate between FA and antisymmetry.

FA in size was estimated as the average between replicates of the absolute differences between centroid of left and right sides for each specimen [15]. FA in shape was computed with MorphoJ [35] as the Procrustes distance, i.e., as the absolute shape differences and deviations from the sample mean, regardless of their direction [7]. Although shape FA can also be estimated as a Mahalanobis distance [7, 40], this approach could not be pursued herein because it requires a larger sample size [40]. Given that any eventual correlation between size and shape may arise from a direct developmental link between these variables, we additionally tested for an allometric relationship between size and shape using multivariate regression [7, 9].

To test the hypothesis that the same mechanism is buffering against perturbations independently form their origin, we used two different methods [12]. First, we used correlation analyses to address each our three research questions [7, 12]:

1. Is there common developmental buffering capacity acting against developmental stability and canalization? To test this hypothesis, we estimated the correlation between inter-individual (individual) and intra-individual variation for shape and size for dorsal and ventral anchors independently.
2. Do mechanisms buffering against variation in shape differ from those acting on size? This question was addressed by estimating the correlations between shape and size for inter-individual and intra-individual variation in each of the anchor pairs (dorsal and ventral).
3. Do buffering mechanisms act differently between dorsal and ventral anchors? To answer this question, we computed the correlations between intra-individual shape, intra-individual size, inter-individual shape and inter-individual size across anchors.

The second method could only be applied to (a) and partly to (c) above, as it is intended for shape-shape comparisons. Congruence of landmark displacements was evaluated by comparison of two PCAs displayed by different sources of variation in the Procrustes ANOVA [14, 34, 41] and specifically to test whether the buffering mechanisms have the same morphological effects on variation [12]. To address question (a), we tested the statistical significance of the angles between the corresponding principal components (PCs) from inter-individual and intra-individual PCAs. We compared the angles between PCs with angles between pairs of random vectors in 2·*LM*– 4 dimensions, where *LM* is the number of landmarks and semilandmarks [6, 12, 14, 34]. This analysis is analogous to correlation analysis among univariate features [6]. In this way, we can address our specific goals by means of correlations and by means of comparing PCs. The same procedure was used to evaluate differences in inter- and intra-individual variation in shape between dorsal and ventral anchors (see (c) above).

## Results

Both, Procrustes ANOVA (shape analysis) and two-way ANOVA (size analysis) revealed a significant effect of the interaction between individual and side, whereas the effect of side was not significant. This result was observed in the analysis of both the dorsal and ventral anchors (Tables 1 and 2). The PCA plot and frequency distributions of R-L differences showed no evidence of antisymmetry in shape and size respectively (S1 and S2 Figs). The mean and standard deviation FA estimates of all analyses are shown in Table 3 and the individual FA values of all analyses are shown in S1 Table.

**Table 1:** Results of Procrustes ANOVA of shape for (A) dorsal and (B) ventral anchors

**A**						
**Factor**	**SS**	**Explained SS (%)**	**MS**	**df**	**F**	***P***
**Individual**	0.15	49.7	8.3·10^−5^	1824	2.5	<0.0001
**Side**	0.00	0.5	1.9·10^−5^	76	0.6	0.99
**Individual × side**	0.06	19.9	3.3·10^−5^	1824	1.4	<0.0001
**Replicate**	0.09	29.9	2.4·10^−5^	3800		
**B**						
**Factor**	**SS**	**Explained SS (%)**	**MS**	**df**	**F**	***P***
**Individual**	0.19	48.5	8.7·10^−5^	2204	1.8	<0.0001
**Side**	0.00	0.7	3.6·10^−5^	76	0.7	0.96
**Individual × side**	0.12	27.6	5.0·10^−5^	2204	2.5	<0.0001
**Replicate**	0.09	23.2	2.0·10^−5^	4560		

SS, sums-of-squares; explained SS (%); MS, mean square; df, degrees of freedom; F, F statistic; *P*, associated probability level.

**Table 2:** Results of two-way ANOVA of size for (A) dorsal and (B) ventral anchors.

**A**						
**Factor**	**SS**	**Explained SS (%)**	**MS**	**df**	**F**	***P***
**Individual**	841	68.7	35.0	24	3.5	0.0016
**Side**	4	0.3	3.8	1	0.4	0.54
**Individual × side**	240	19.6	10.0	24	3.6	<0.0001
**Replicate**	141	11.5	2.8	50		
**B**						
**Factor**	**SS**	**Explained SS (%)**	**MS**	**df**	**F**	***P***
**Individual**	1175	69.3	40.5	29	2.8	0.0032
**Side**	0.2	0.0	0.2	1	0.0	0.91
**Individual × side**	414	24.4	14.3	29	8	<0.0001
**Replicate**	107	6.3	1.8	60		

SS, sums-of-squares; explained SS (%); MS, mean square; df, degrees of freedom; F, F statistic; *P*, associated probability level.

**Table 3:** Mean estimates of fluctuating asymmetry (± standard deviation) in shape and size of dorsal and ventral anchors.

Anchor	x ± SD
Dorsal (shape)	0.05 ± 0.01
Ventral (shape)	0.06 ± 0.02
Dorsal (size)	2.49 ± 1.95
Ventral (size)	3.05 ± 2.15

SD, standard deviation; x, FA mean value.

The results of the implemented submodels without the interaction term agree with the results of the full model (with the interaction term). The submodels showed a significant effect of the individual factor for shape and size analyses for both dorsal and ventral anchors. The side effect was not significant (S2 and S3 Tables). In reference to the normality assumption of the two-way ANOVA, residuals were normally distributes (*P >* 0.18 for both types of anchors). There was no evidence for violations of homoscedasticity for both Procrustes (*P >* 0.14 for both types of anchors) and two-way ANOVA (*P >* 0.22 for both types of anchors).

**Fig 2:**
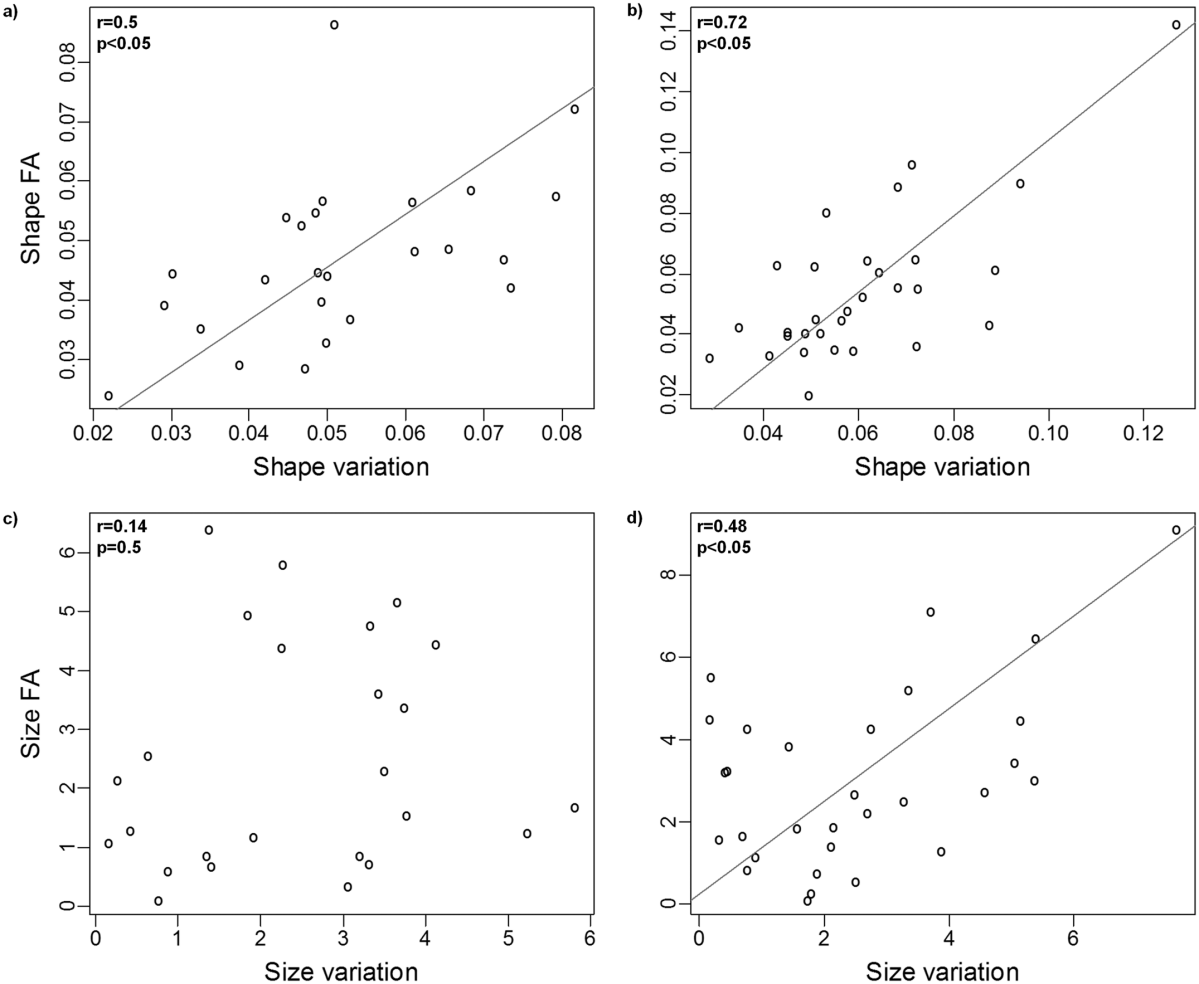
Relationship between anchors inter- and intra-individual variation. Relationship between inter- and intra-individual variation (Fluctuating asymmetry, FA) of shape (A, B) and size (C, D) in dorsal (A, C) and ventral (B, D) anchors. Trend lines are standard major axis regressions, (shown only in case of significant correlation between the variables of interest).

**Fig 3:**
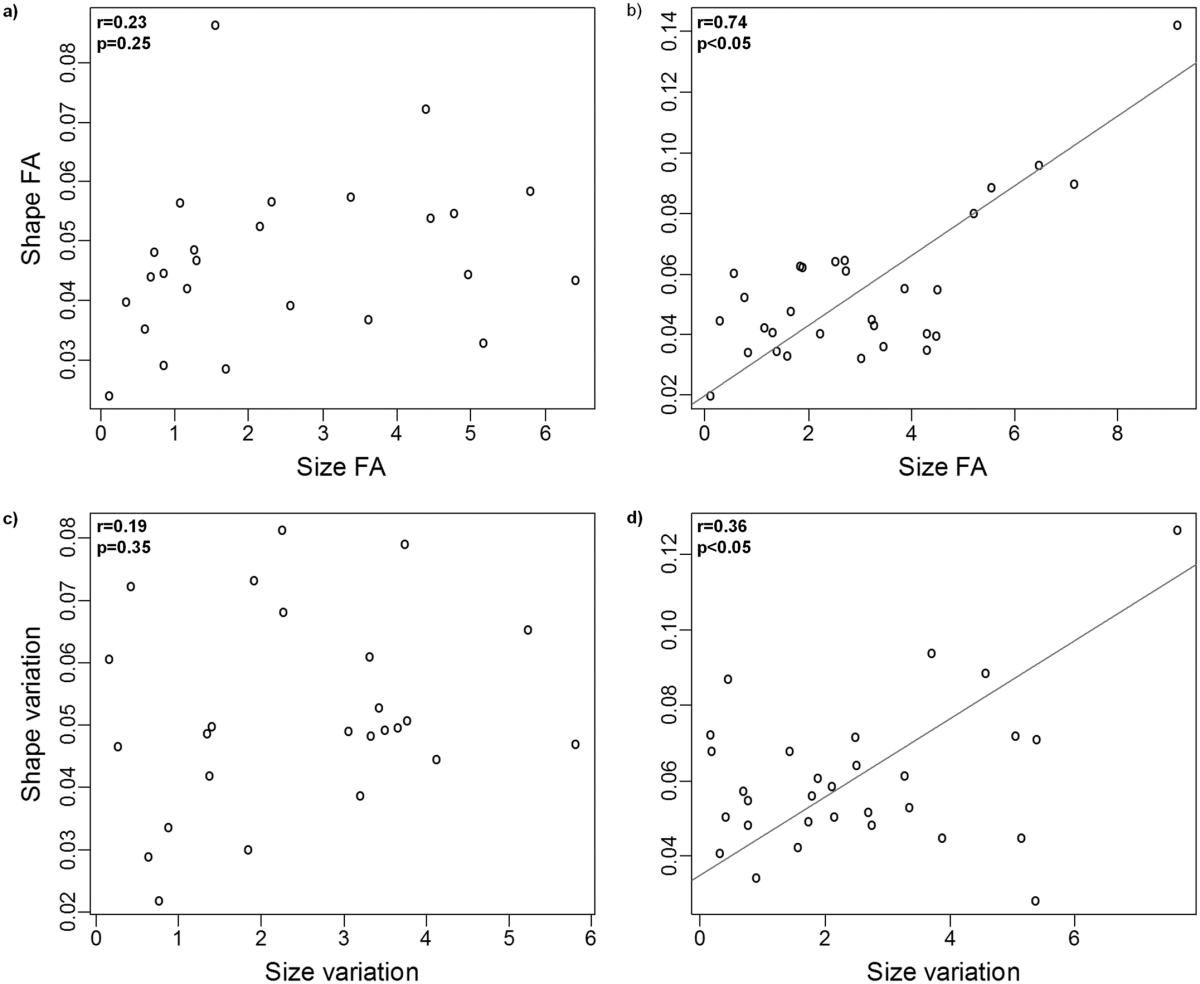
Relationship between anchors size and shape variation. Relationship between size and shape intra-individual (Fluctuating asymmetry, FA) (A, B) and inter-individual (C, D) variation in dorsal (A, C) and ventral (B, D) anchors. Trend lines are standard major axis regressions, (shown only in case of significant correlation between the variables of interest).

**Fig 4:**
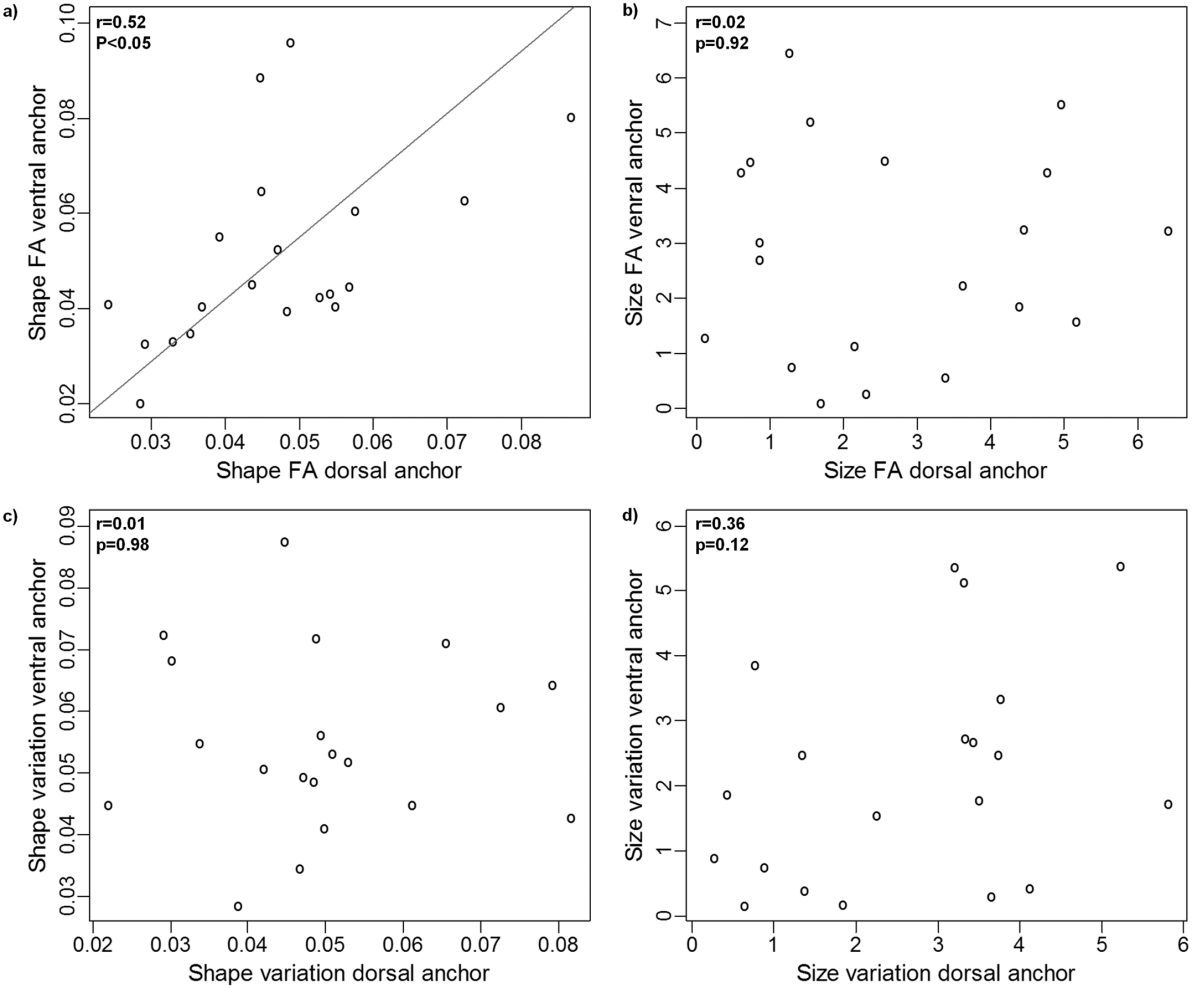
Relationship between dorsal and ventral anchors. Relationship between dorsal and ventral anchors in intra-individual shape (A) and size (B) variation and in inter-individual shape (C) and size (D) variation. Trend lines are standard major axis regressions, (shown only in case of significant correlation between the variables of interest).

There was a significant allometric relationship between size and shape in both dorsal and ventral anchors (*P*< 0.0001 in both cases). However, size accounted for a small fraction of size variation in dorsal and ventral anchors (6.5% and 7.7%, respectively). Inter- and intra-individual (FA) variation in shape were significantly correlated in both dorsal and ventral anchors. As for size, the relationship between inter- and intra-individual variation was only significant in the ventral anchors (Fig 2). Likewise the correlation between intra-individual (FA) shape and size, and between inter-individual variation in shape and size was only significant in the ventral anchors (Fig 3). Only intra-individual (FA) variation in shape was significantly correlated in ventral and dorsal anchors (Fig 4).

**Fig 5:**
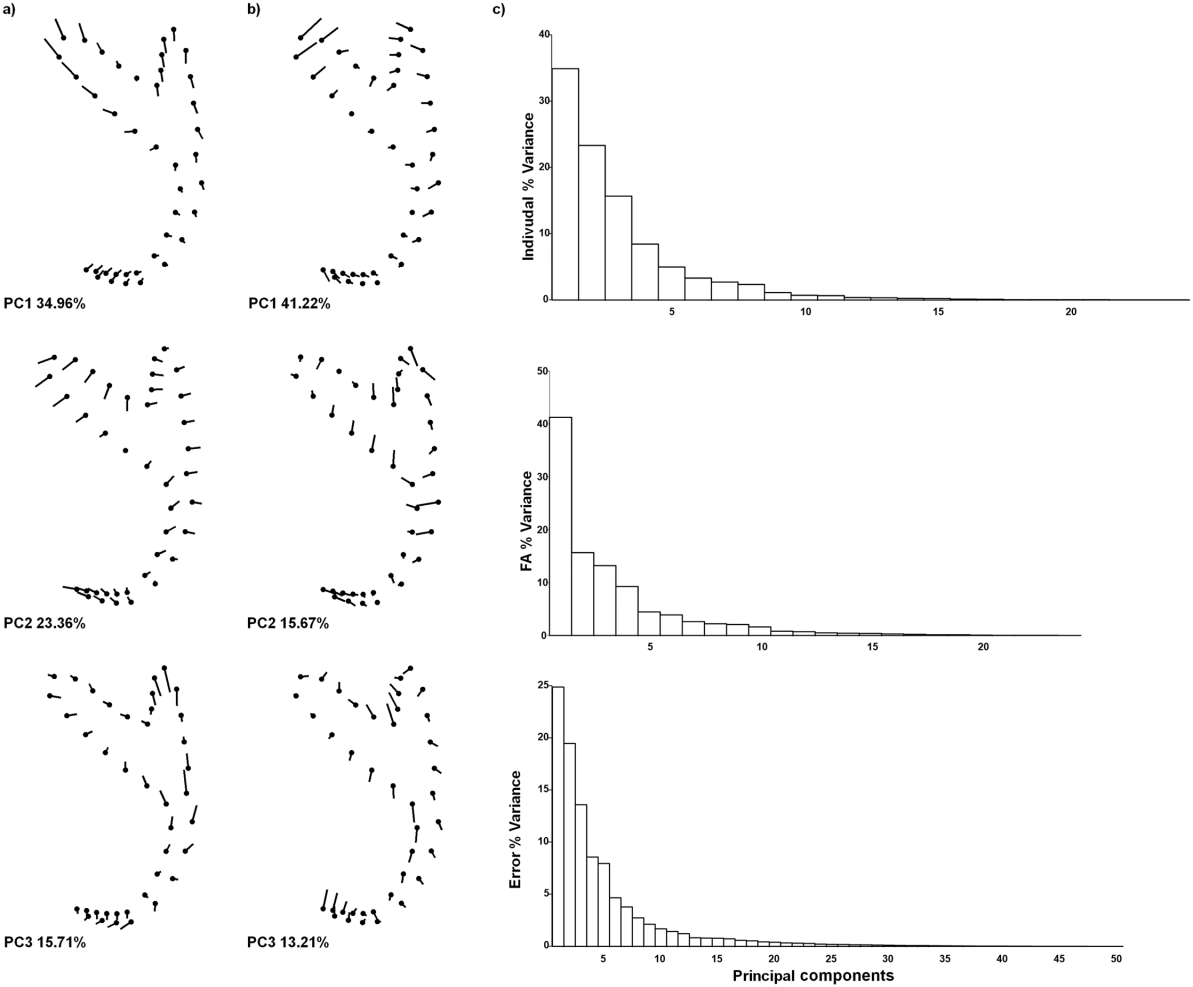
Lollipops graphs of Principal Component Analysis of the dorsal anchors. (A) Inter- and (B) intra-individual variation (Fluctuating asymmetry, FA). Graphs display the landmarks displacements for the first three principal components (PCs) of Principal component analyses of factors in the Procrustes ANOVA. (C) Percentage of shape variation explained by each PC in the different analyses.

**Fig 6:**
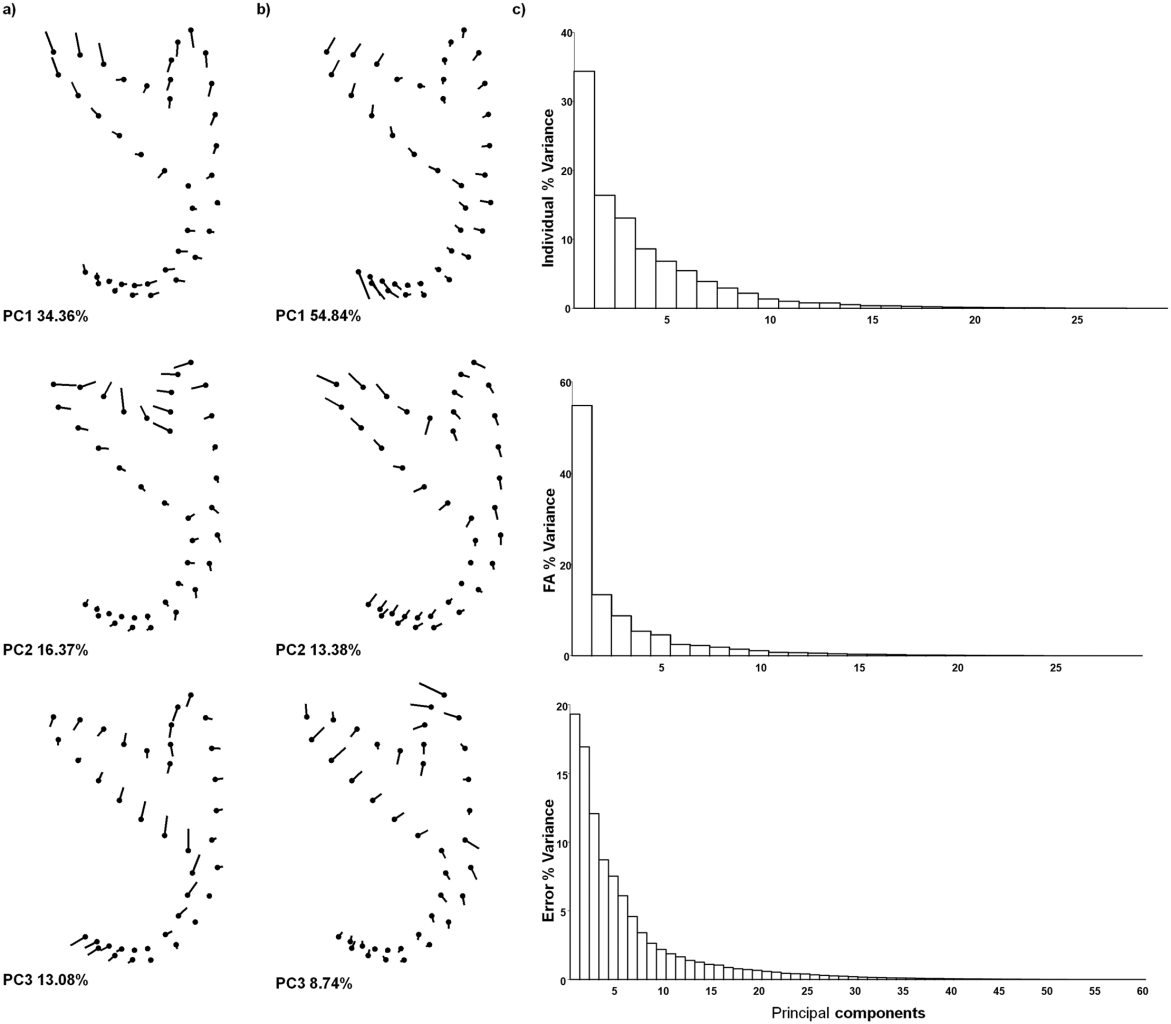
Lollipops graphs of Principal Component Analysis of the ventral anchors. (A) Inter- and (B) intra-individual variation (Fluctuating asymmetry, FA). Graphs display the landmarks displacements for the first three principal components (PCs) of Principal component analyses of factors in the Procrustes ANOVA. (C) Percentage of shape variation explained for each PC in the different analyses.

The landmark displacements are graphically represented as lollipop graphs (Figs 5 and 6). Broadly, most shape variation affected the root lengths and, in some dimensions, the point orientation. In the dorsal anchors (Fig 5), the first three PCs explained 74.0% of the total variance for inter-individual variation; 70.1% for intra-individual variation; and 58.0% for measurement error. In the ventral anchors (Fig 6), the first three PCs accounted for 63.8%, 77.0% and 48.3% of the total variance for inter-individual, intra-individual variation and measurement error, respectively. The angular tests revealed that patterns of morphological variation in anchor shape were in general coherent, because angle between PCs were significantly smaller than angles between pairs of random vectors. These results were obtained for both comparisons between inter- (individual variation) and intra-individual (FA) variation, and between dorsal and ventral anchors (Table 4). When the angular analysis is significant, coordinate landmark displacement between PCs can be compared by the visual inspection of lollipop graphs (Figs 5 and 6).

**Table 4:** Angles (°) between principal components of landmark displacements and significance levels of the comparisons.

	I vs. FA		D vs. V	
PC	D	V	I	FA
**1**	87.61	76.21**	31.12***	50.86***
**2**	74.42*	69.11***	66.88***	87.33
**3**	72.66**	89.25	61.13***	84.51

PC: principal component; I: inter-individual variation; FA: intra-individual variation (fluctuating asymmetry); D: dorsal anchors; V: ventral anchor.

Significance levels:

*P ≤ 0.05;

**P ≤ 0.01;

***P ≤ 0.001.

## Discussion

Monogeneans show definite patterns of symmetry in their haptoral structures, but few studies have attempted to quantify such patterns [23]. One of the scarce instances is Pečínková et al. [42], in that classical morphometrics were applied to the anchors of *Paradiplozoon homoion* Bychowsky et Nagibina, 1959 to document the occurrence of FA and directional asymmetry in different traits. Other researches have employed GM to study shape and size variation of haptoral structures and their relation with ecology and phylogeny [20, 23, 29]. However, as far as we know, no previous study has approached the quantitative study of the symmetry of the anchors of monogeneans using the power and versatility provided by GM techniques and we provide herein for the first time evidence for FA in a monogenean using GM.

Although we found an allometric relationship between size and shape in both anchor types, the percentage of shape variation directly accounted by size was quite small in both cases. It is therefore likely that the correlations between shape and size reported herein are not simply the result of a direct allometric link between these variables. Accordingly, our correlation results suggests that the two processes responsible of ensuring phenotypic constancy, canalization and developmental stability [6, 14], act on the same components of shape of both types of anchors and size of ventral anchors. This supports the notion of a general developmental mechanism buffering all perturbations independently from their origin, which is in line with previous carefully controlled empirical studies [13] acting on anchor shape and on size of the ventral anchors. Because in monogeneans survival depends critically on efficient anchoring to the host [20, 43–45], anchor morphology probably represents essential components of fitness likely subjected to homeostatic development [6, 46]. Therefore, our results in favour of a common developmental buffering capacity are perhaps not surprising, given that high congruence between inter- and intra-individual morphological variation is expected when the character under study is representative of individual fitness [6].

Interestingly all correlations involving either inter- and intra-individual variation in size of dorsal anchors were not significant. Thus, size variation of dorsal anchors was much less predictable than that of the ventral anchors. In addition, inter-individual shape variation of the dorsal anchors was not significantly correlated with that of ventral anchors. However, the position of inter-individual landmarks varied consistently between dorsal and ventral anchors across individuals. Although this may seem contradictory, note that each analysis tested different, albeit related, hypotheses. The result of the correlation analysis indicates that individuals with dorsal anchors that most deviate from the average dorsal anchor shape, do not necessarily exhibit ventral anchors than most deviate from the average ventral shape and vice versa. In contrast, the angular analysis tests for congruence in the position of individual landmarks and visual inspection of Figs 5A and 6A suggests concerted localized variation of the roots and point. In other words, the difference between the two tests is probably due to the anisotropic variation of landmarks positions among individuals [40]. This concerted localized variation conforms to the high integration in shape between the ventral and dorsal anchors reported in *L*. *cephali* [21].

Although the specific mechanisms governing developmental buffering are still unclear [6, 9], the evidence brought forward herein suggests different strengths on buffering variation of ventral and dorsal anchors and on buffering size and shape. This supports the idea that traits evolve independently and that the phenotypic effect of changes in signalling strength may be non-lineal and responding to thresholds [13]. Further studies comparing size and shape between dorsal and ventral haptoral structures in dactylogyrids are needed. Note also that this approach could be further extended and generalized to a number of Polyopisthocotylea where the morphology of right and left clamps could be compared serially.

**Fig 7:**
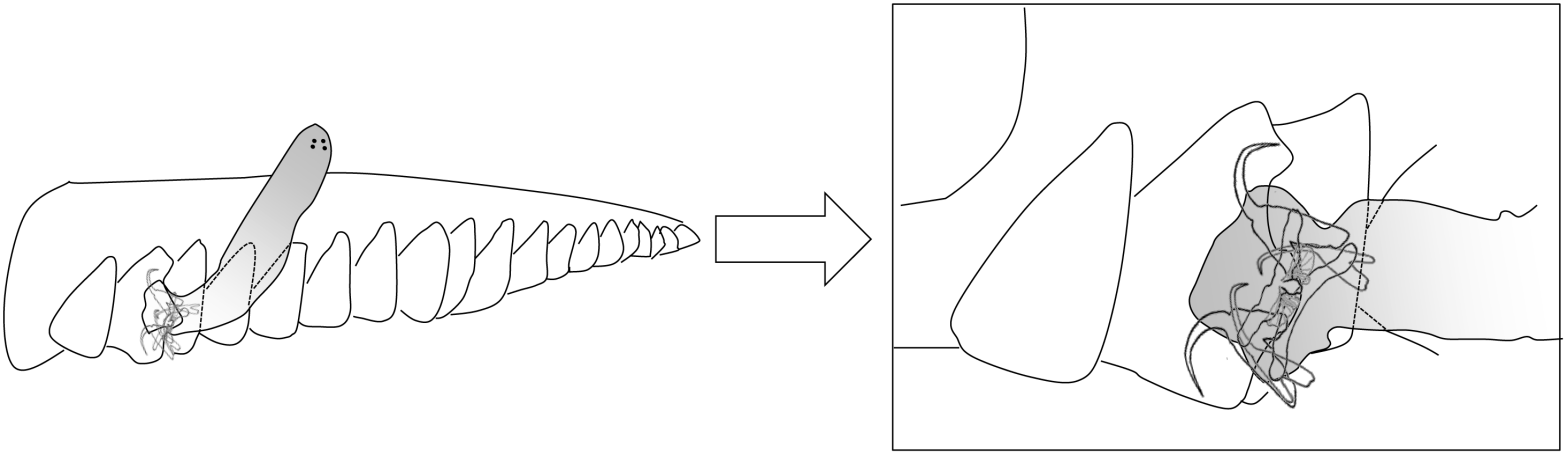
Diagrammatic representation of the attachment strategy of *Ligophorus cephali*. *Ligophorus cephali* specimen attached to secondary lamella of a gill filament. Modified from Sánchez-García et al. [47].

Interspecific differences between dorsal and ventral anchors and bars morphologies have been examined in *L*. *cephali* and other Dactylogyridae [21, 25, 28]. Rodríguez-González et al. [21] quantified the phenotypic plasticity of the anchors in *L*. *cephali* and their results indicated a tighter control of the shape and size of the ventral anchors, which fully agrees with the present evidence. The authors proposed that the different levels of morphological variation between ventral and dorsal anchors reflect their different functional roles. The former seemed the most important for attachment and would respond more closely in size and shape to individual host characteristics [21]. Other studies have explicitly explained the role played by the different pieces of the haptor [44, 47, 48] and they agree with a different functionality of dorsal and ventral anchors. Initially, anchors spread the gill filament [44, 47] and the points penetrate into the epithelium [47]. When the parasite is attached, ventral anchors distribute the force over a larger area whereas dorsal anchors rest in a pincer-like position [44, 48] (Fig 7). In relation to our findings, it can be argued that developmental buffering in dorsal anchors is weaker because their morphological variation does not compromise their functional role in attachment.

The present study exemplifies how to obtain statistical evidence for the existence of buffering mechanisms on phenotypic variation. Estimates of FA in both shape and size can be further used to evaluate differences between populations or to model the magnitude or direction of phenotypic changes associated to environmental or genetic stress [49]. However, controlled experiments would be required to partition the genetic, micro- and macro-environmental components of phenotypic variation [6, 7, 41, 50]. Gyrodactylids in particular would provide a convenient model for experiments aimed at isolating the genetic and environmental components of canalization because genetic variation can be minimized by means of laboratory-reared isogenic lineages [51]. This approach could be particularly useful in a variety of settings, for instance, to quantify the influence of physiochemical parameters [52], geographical origin [20] or time variables [49]. Similarly, although several studies have questioned the relationship between FA and fitness [3, 53], direct and indirect correlation between these two terms is well documented [17, 18, 46, 52]. In aquaculture, haptoral symmetry may be taken to reflect environmental stress, affording to experimentally evaluate, for instance, the effectiveness of control treatments.

In summary, the present study suggests that the effect of buffering is more evident on the morphological anchoring elements that seem more important for attachment. Accordingly, we hypothesize that homeostatic development of these elements is promoted to ensure the morphological constancy required for efficient attachment. We believe that further studies of phenotypic buffering in ectoparasites, testing this and allied hypotheses, have potential to provide valuable insights into the evolutionary ecology of parasitism.

## Supporting Information

S1 Fig Principal component analyses of raw coordinates. (A) Dorsal and (B) ventral anchors. Both analyses showed a single cluster of points that includes right (red points) and left (blue) revealing the existence of fluctuating asymmetry (FA) for shape.
(TIF)

S2 Fig Frequencies distribution of signed Right-Left (R-L) differences of size. (A) Dorsal and (B) ventral anchors. The unimodal distributions were indicative of fluctuating asymmetry (FA) for size.
(TIF)

S1 Table Fluctuating asymmetry (FA) values estimated for size and shape of dorsal and ventral anchors. (DOC)

S2 Table Results of Procrustes ANOVA without the interaction term of shape for (A) dorsal and (B) ventral anchors. (DOC)

S3 Table Results of two-way ANOVA without the interaction term of shape for (A) dorsal and (B) ventral anchors. (DOC)
