# Supplementary figures and images for: Phenotypic Buffering in a Monogenean: Canalization and Developmental Stability in Shape and Size of the Haptoral Anchors of Ligophorus cephali (Monogenea: Dactylogyridae)
Source: PLoS One. 2015 Nov 6;10(11):e0142365. doi: 10.1371/journal.pone.0142365 (PMC4636253; doi:10.1371/journal.pone.0142365)

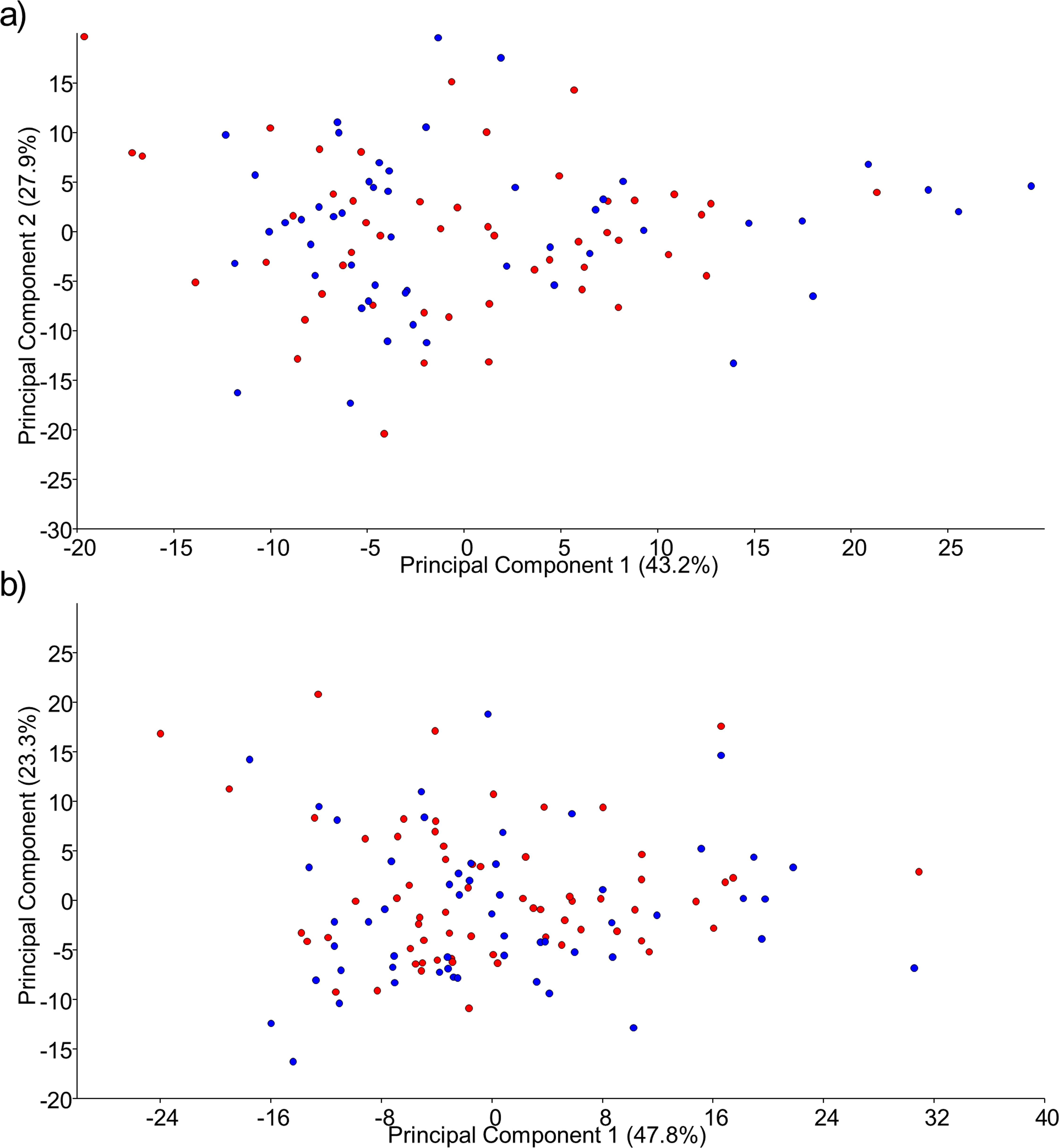

Supplement: S1 Fig — (A) Dorsal and (B) ventral anchors. Both analyses showed a single cluster of points that includes right (red points) and left (blue) revealing the existence of fluctuating asymmetry (FA) for shape. (TIF) [file pone.0142365.s001.tif]
