# Supplementary material for: Phenotypic Buffering in a Monogenean: Canalization and Developmental Stability in Shape and Size of the Haptoral Anchors of Ligophorus cephali (Monogenea: Dactylogyridae)
Source: PLoS One. 2015 Nov 6;10(11):e0142365. doi: 10.1371/journal.pone.0142365 (PMC4636253; doi:10.1371/journal.pone.0142365)
Supplement: S1 Table — (DOC) [file pone.0142365.s003.doc]

**S1** Table. Fluctuating asymmetry (FA) values estimated for size and shape of dorsal and ventral anchors.

|  | **Dorsal** | | **Ventral** | |
| --- | --- | --- | --- | --- |
| **Id** | **Shape** | **Size** | **Shape** | **Size** |
| **Ligo002** | 0.05 | 1.30 | 0.05 | 0.76 |
| **Ligo003** | - | - | 0.14 | 9.14 |
| **Ligo005** | 0.05 | 4.77 | 0.04 | 4.29 |
| **Ligo006** | 0.02 | 0.12 | 0.04 | 1.30 |
| **Ligo007** | 0.04 | 3.63 | 0.04 | 2.24 |
| **Ligo008** | 0.04 | 1.18 | - | - |
| **Ligo009** | 0.05 | 4.46 | 0.04 | 3.27 |
| **Ligo010** | 0.04 | 2.56 | 0.06 | 4.51 |
| **Ligo011** | 0.05 | 2.16 | 0.04 | 1.15 |
| **Ligo012** | 0.04 | 0.35 | - | - |
| **Ligo013** | - | - | 0.03 | 0.84 |
| **Ligo015** | - | - | 0.04 | 3.46 |
| **Ligo016** | - | - | 0.06 | 2.74 |
| **Ligo017** | 0.03 | 0.86 | 0.03 | 3.02 |
| **Ligo018** | 0.06 | 2.31 | 0.04 | 0.28 |
| **Ligo021** | 0.04 | 6.41 | 0.05 | 3.23 |
| **Ligo022** | 0.05 | 0.73 | 0.04 | 4.48 |
| **Ligo023** | 0.04 | 0.86 | 0.06 | 2.70 |
| **Ligo024** | 0.03 | 1.69 | 0.02 | 0.11 |
| **Ligo029** | - | - | 0.06 | 2.52 |
| **Ligo032** | 0.06 | 3.38 | 0.06 | 0.57 |
| **Ligo033** | - | - | 0.06 | 3.87 |
| **Ligo034** | 0.06 | 1.09 | - | - |
| **Ligo035** | - | - | 0.06 | 1.90 |
| **Ligo037** | 0.05 | 1.26 | 0.10 | 6.47 |
| **Ligo045** | - | - | 0.03 | 1.40 |
| **Ligo048** | 0.07 | 4.40 | 0.06 | 1.86 |
| **Ligo060** | 0.04 | 0.61 | 0.04 | 4.29 |
| **Ligo061** | 0.03 | 5.17 | 0.03 | 1.59 |
| **Ligo066** | 0.06 | 5.81 | - | - |
| **Ligo068** | 0.04 | 0.69 | - | - |
| **Ligo087** | 0.04 | 4.96 | 0.05 | 1.66 |
| **Ligo091** | 0.04 | 4.96 | 0.09 | 5.54 |
| **Ligo093** | - | - | 0.09 | 7.15 |
| **Ligo094** | 0.09 | 1.56 | 0.08 | 5.22 |
