# Supplementary material for: Phenotypic Buffering in a Monogenean: Canalization and Developmental Stability in Shape and Size of the Haptoral Anchors of Ligophorus cephali (Monogenea: Dactylogyridae)
Source: PLoS One. 2015 Nov 6;10(11):e0142365. doi: 10.1371/journal.pone.0142365 (PMC4636253; doi:10.1371/journal.pone.0142365)
Supplement: S2 Table — (DOC) [file pone.0142365.s004.doc]

**S2 Table. Results of Procrustes ANOVA without interaction of shape for (A) dorsal and (B) ventral anchors.**

(A)

| **Factor** | **SS** | **Explained SS (%)** | **MS** | **df** | **F** | ***P*** |
| --- | --- | --- | --- | --- | --- | --- |
| **Individual** | 0.151 | 49.8 | 0.00011 | 1824 | 4 | <0.0001 |
| **Side** | 0.001 | 0.5 | 1.3·10^-5^ | 76 | 0.5 | 0.99 |
| **Replicate** | 0.151 | 49.8 | 2.7·10^-5^ | 5624 |  |  |

(B)

| **Factor** | **SS** | **Explained SS (%)** | **MS** | **df** | **F** | ***P*** |
| --- | --- | --- | --- | --- | --- | --- |
| **Individual** | 0.192 | 48.5 | 8.7·10^-5^ | 2204 | 3 | <0.0001 |
| **Side** | 0.002 | 0.49 | 2.6·10^-5^ | 76 | 0.9 | 0.71 |
| **Replicate** | 0.202 | 51.01 | 2.9·10^-5^ | 6764 |  |  |

SS, sums-of-squares; explained SS (%); MS, mean square; df, degrees of freedom; F, F statistic; *P*, associated probability level.
