# Supplementary material for: Phenotypic Buffering in a Monogenean: Canalization and Developmental Stability in Shape and Size of the Haptoral Anchors of Ligophorus cephali (Monogenea: Dactylogyridae)
Source: PLoS One. 2015 Nov 6;10(11):e0142365. doi: 10.1371/journal.pone.0142365 (PMC4636253; doi:10.1371/journal.pone.0142365)
Supplement: S3 Table — (DOC) [file pone.0142365.s005.doc]

**S3 Table. Results of two-way ANOVA without interaction of size for (A) dorsal and (B) ventral anchors.**

(A)

| **Factor** | **SS** | **Explained SS (%)** | **MS** | **df** | **F** | ***P*** |
| --- | --- | --- | --- | --- | --- | --- |
| **Individual** | 841.2 | 68.6 | 35.05 | 24 | 6.8 | <0.0001 |
| **Side** | 3.8 | 0.4 | 3.84 | 1 | 0.74 | 0.39 |
| **Replicate** | 380.2 | 31 | 5.14 | 74 |  |  |

(B)

| **Factor** | **SS** | **Explained SS (%)** | **MS** | **df** | **F** | ***P*** |
| --- | --- | --- | --- | --- | --- | --- |
| **Individual** | 1175 | 69.2 | 40.5 | 29 | 6.9 | <0.0001 |
| **Side** | 0.7 | 0.2 | 0.75 | 1 | 0.12 | 0.72 |
| **Replicate** | 520.6 | 30.6 | 5.85 | 89 |  |  |

SS, sums-of-squares; explained SS (%); MS, mean square; df, degrees of freedom; F, F statistic; *P*, associated probability level.
